# Analysis of bronchoalveolar lavage fluid metatranscriptomes among patients with COVID-19 disease

**DOI:** 10.1038/s41598-022-25463-0

**Published:** 2022-12-07

**Authors:** Michael Jochum, Michael D. Lee, Kristen Curry, Victoria Zaksas, Elizabeth Vitalis, Todd Treangen, Kjersti Aagaard, Krista L. Ternus

**Affiliations:** 1 Division of Maternal-Fetal Medicine, Department of Obstetrics and Gynecology, Baylor College of Medicine, Texas Children’s Hospital, Houston, TX 77030 USA; 2 Blue Marble Space Institute of Science, Seattle, WA 98104 USA; 3 Department of Computer Science, Rice University, Houston, TX 77005 USA; 4 Center for Translational Data Science, University of Chicago, Chicago, IL 60615 USA; 5 Clever Research Lab LLC, 2501 Chatham Rd, Suite N, Springfield, IL 62704 USA; 6 Inscripta, Inc, 5500 Central Ave STE 220, Boulder, CO 80301 USA; 7 Signature Science, LLC, 8329 North Mopac Expressway, Austin, TX 78759 USA

**Keywords:** Classification and taxonomy, Functional clustering, Gene ontology, Microbial communities

## Abstract

To better understand the potential relationship between COVID-19 disease and hologenome microbial community dynamics and functional profiles, we conducted a multivariate taxonomic and functional microbiome comparison of publicly available human bronchoalveolar lavage fluid (BALF) metatranscriptome samples amongst COVID-19 (*n* = 32), community acquired pneumonia (CAP) (*n* = 25), and uninfected samples (*n* = 29). We then performed a stratified analysis based on mortality amongst the COVID-19 cohort with known outcomes of deceased (*n* = 10) versus survived (*n* = 15). Our overarching hypothesis was that there are detectable and functionally significant relationships between BALF microbial metatranscriptomes and the severity of COVID-19 disease onset and progression. We observed 34 functionally discriminant gene ontology (GO) terms in COVID-19 disease compared to the CAP and uninfected cohorts, and 21 GO terms functionally discriminant to COVID-19 mortality (q < 0.05). GO terms enriched in the COVID-19 disease cohort included hydrolase activity, and significant GO terms under the parental terms of biological regulation, viral process, and interspecies interaction between organisms. Notable GO terms associated with COVID-19 mortality included nucleobase-containing compound biosynthetic process, organonitrogen compound catabolic process, pyrimidine-containing compound biosynthetic process, and DNA recombination, RNA binding, magnesium and zinc ion binding, oxidoreductase activity, and endopeptidase activity. A Dirichlet multinomial mixtures clustering analysis resulted in a best model fit using three distinct clusters that were significantly associated with COVID-19 disease and mortality. We additionally observed discriminant taxonomic differences associated with COVID-19 disease and mortality in the genus *Sphingomonas,* belonging to the Sphingomonadacae family, *Variovorax,* belonging to the Comamonadaceae family, and in the class Bacteroidia*,* belonging to the order Bacteroidales. To our knowledge, this is the first study to evaluate significant differences in taxonomic and functional signatures between BALF metatranscriptomes from COVID-19, CAP, and uninfected cohorts, as well as associating these taxa and microbial gene functions with COVID-19 mortality. Collectively, while this data does not speak to causality nor directionality of the association, it does demonstrate a significant relationship between the human microbiome and COVID-19. The results from this study have rendered testable hypotheses that warrant further investigation to better understand the causality and directionality of host–microbiome–pathogen interactions.

## Introduction

Metatranscriptomes from tissues and biologic samples arising from hosts with varying disease severity and outcomes represent a rich source of information to evaluate the role of the microbiome in onset and progression. For respiratory viruses like SARS-CoV-2, bronchoalveolar lavage fluid (BALF) is a valuable sample type collected to investigate the biology of lower respiratory tract infections. Unfortunately, this sample type is more challenging to obtain for research studies that require large numbers of matching cases and controls, especially compared to the more easily accessible sample types like nasopharyngeal swabs. In general, BALF samples arise from patients that either have a clinical indication for them to be obtained or from healthy controls that have consented for the procedure. Early in the SARS-CoV-2 outbreak, scientists published metatranscriptome sequences from BALF of patients with COVID-19 disease and made the data available in the public domain (Suppl. Table 1); however, limitations in the sample numbers and lack of uniformity in study designs across different laboratories prevented a robust statistical analysis from taking place. In this paper, we computationally evaluate microbial insights drawn from these valuable BALF samples, despite the experimental study design limitations. In contrast to other studies that focus on characteristics of the human host response or SARS-CoV-2 lineages and viral variants, our analysis specifically evaluated the microbial taxonomic and functional profiles of the BALF metatranscriptomes. The role of the human microbiome in SARS-CoV-2 infection is poorly understood, but it remains important to study, since it could be a significant contributor to the observed variations in COVID-19 disease severity and resiliency between patients.

Among other risk factors, it is possible that the lower respiratory tract microbiome plays a role in COVID-19 disease severity. A previous 16S rRNA gene study found that COVID-19 patient endotracheal aspirates had lower microbial diversity compared to uninfected individuals, but these differences were not found to have a significant impact on fatality outcomes^1^. The original Shen et al. study^2^ performed a microbial taxonomic analysis of sequenced BALF metatranscriptomes without evaluating functional profiles or considering COVID-19 disease severity in the microbial analysis. Haiminen et al.^3^ reanalyzed BALF metatranscriptome sequences from the Shen et al. study^2^ and identified differences in expressed metabolic pathways in COVID-19 samples compared to the uninfected and community acquired pneumonia (CAP) cohorts; however, functional profile differences were not analyzed based on COVID-19 clinical severity. Yang et al.^4^ analyzed previously published BALF metatranscriptome datasets from multiple independent studies^2,5–10^ and performed a comparative taxonomic analysis between samples from COVID-19 patients and healthy control groups but did not subdivide cohorts further or perform functional analyses. Other studies have focused solely on the taxonomic analysis of a subset of published BALF metatranscriptomes and specific potential co-infections that may be present^11,12^. To our knowledge, this study is the first to evaluate significant differences in taxonomic and functional signatures between BALF metatranscriptomes from COVID-19, CAP, and uninfected cohorts, as well as COVID-19 morbidity and mortality.

To better understand the potential relationship between COVID-19 morbidity and mortality and the human-microbiome, we conducted an analysis using human BALF metatranscriptome samples sourced from eight publications and nine corresponding public data repositories (Suppl. Tables 1 and 2). BALF specimens from individual subjects were grouped into one of three categorical classes: (1) uninfected controls; (2) community acquired pneumonia (CAP) patients; or (3) COVID-19 patients with moderate to severe disease, including death (Table 1). The objectives of the current study were to compare the BALF metatranscriptomes amongst and between each of the three cohort categorical classes or their sub-categories, such as COVID-19 severe disease versus death, and to identify significantly associated taxonomic and functional differences in microbial derived community dynamics. To achieve these objectives, relevant metatranscriptome datasets were compiled from public sources and a rigorous analysis pipeline was implemented to assess (1) the composition of the microbiome taxa in association with respiratory disease and (2) the microbial gene functions significantly perturbed.

**Table 1:** Overview of meta-analysis dataset clinical characteristics (*n* = 86).

**Variable**	Uninfected	Community acquired pneumonia	COVID-19
**Cohort**	29 (33.72%)	25 (29.07%)	32(37.21%)
**Outcome (COVID-19 only)**			
Deceased	–	–	10 (31.25%)
Survived	–	–	15 (46.87%)
Unspecified	–	–	7 (21.88%)
**Sex**			
Female (n = 22)	4 (18.18%)	8 (36.36%)	10 (45.45%)
Male (n = 38)	5 (13.15%)	11 (28.94%)	22 (57.89%)
Unspecified (n = 26)	20 (76.92%)	6(23.07%)	0 (0%)
**Reads**			
Paired	29 (37.18%)	25 (32.05%)	24 (30.77%)
Single	0 (0%)	0 (0%)	8 (100%)
**Publication**			
Chen	0 (0%)	0 (0%)	2 (100%)
Ren	9 (100%)	0 (0%)	0 (0%)
Shen	20 (32.79%)	25 (40.98%)	16 (40.98%)
Wu	0 (0%)	0 (0%)	1 (100%)
Xiong	0 (0%)	0 (0%)	4 (100%)
Zhou	0 (0%)	0 (0%)	9 (100%)
**Numeric variables (mean ± SD)**			
Age	53.2 ± 13.3 (*n* = 9)	51.2 ± 19.8 (*n* = 17)	47.3 ± 11.5 (*n* = 32)
Temp. °C	–	38.4 ± 0.91 (*n* = 15)	38.4 ± 0.715 (*n* = 8)
Days after onset	–	9.07 ± 3.17 (*n* = 14)	12.05 ± 6.5 (*n* = 41)

Our overarching testable hypothesis was that there is a potentially informative and discernably significant relationship between the BALF microbiome and the severity of COVID-19 disease. We tested this hypothesis with the following aims: (a) identify significantly associated taxonomic differences between each of the three cohort categorical classes (i.e., uninfected, CAP, COVID-19), (b) discern microbiome-derived functional changes attributed to these community dynamics, and (c) assess these taxonomic and functional differences in relation to the COVID-19 disease outcomes of survived vs. deceased.

## Methods

### Data acquisition and exclusion

Between the beginning of the COVID-19 pandemic and May 2022, we identified five studies with COVID-19 BALF samples and five studies with non-COVID-19 BALF samples (Suppl. Tables 1 and 2)*.* The publicly available Illumina reads were downloaded from the National Center for Biotechnology Information (NCBI) Sequence Read Archive (SRA) or the China National Center for Bioinformation (CNCB) National Genomics Data Center (NGDC), along with the original publications where the clinical information was obtained for downstream analysis of BALF samples^2,5–8,13–15^. Sample types of “unknown” and “sick” from Huang et al.^14^ and Michalovich et al*.*^8^ were pruned from subsequent analysis. “Healthy” samples from Michalovich et al*.*^8^ and SARS-CoV-2 viral-enriched samples from Shen et al.^2^ (PRJNA605907) were also pruned from subsequent analysis (Suppl. Tables 1 and 2). Whenever negative controls were present, the R package decontam^25^ was used to identify and remove potential contaminating organisms. All negative controls (i.e., CRR125995, CRR125996, CRR125997, CRR125998) came from the Shen et al. study, where the negative controls were described in the original publication as either a nuclease-free water sample or saline solutions that passed through the bronchoscope^2^. After read-filtering and batch-effect sample removal, sample cohorts of *n* = 29 uninfected samples from 29 subjects, *n* = 25 CAP samples from 25 subjects, and *n* = 32 COVID-19 samples from 18 subjects were available for comparison (total *n* = 86 BALF samples from *n* = 72 subjects, where a subset of subjects were sampled multiple times). Amongst the COVID-19 cohort at the time of the index study publication, *n* = 10 samples were from 5 known-deceased subjects, *n* = 15 samples were from 9 known-survived subjects, and *n* = 7 from 4 subjects of the total 32 COVID-19 samples in this meta-analysis with unknown / unpublished survival outcomes.

### Quality control and data preprocessing

After the raw reads were downloaded from their sources, the quality of the reads was assessed before and after trimming with FastQC^16^, and quality control (e.g., adapter removal) was performed on the downloaded sequence reads with Trimmomatic^17^. To control for different sequencing approaches by dataset (e.g., datasets being paired, or single-end reads), all paired-end reads were merged with FLASH^18^ and concatenated with unmerged reads into one fastq file per sample. Human and PhiX reads were filtered out with a custom Kraken2^19^ database built with solely human and PhiX references (see data and script availability section below), and low-complexity sequences were removed with fastp^20^.

### Taxonomic and functional assignments

After data preprocessing, a taxonomic analysis was subsequently performed with Kraken2^19^ utilizing their standard database. The processed fastq datasets with human and PhiX reads removed were converted to fasta files and analyzed with SeqScreen^21^ to obtain a list of leaf-node molecular function and biological process Gene Ontology (GO) terms present within each of the samples. The CoV-IRT-Micro conda package (https://github.com/AstrobioMike/CoV-IRT-Micro), along with programs modified from the *bit* package^22^, was used to propagate parent GO terms, parse SeqScreen outputs by taxonomic domain, and summarize Kraken2 taxonomic results and SeqScreen-reported protein identifiers. Parent-propagated GO term counts for all domains other than eukaryotes were imported into a working phyloseq^23^ object alongside collected and curated clinical metadata using R 4.03^24^. GO term abundances from the remaining subjects’ specimens were compositionally transformed, center log ratio (CLR) normalized, and independently compared by case type (COVID-19 vs. CAP and Uninfected) and survival outcome (COVID-19 only deceased vs. survived) via MaAsLin2^26^ using minimum abundance, prevalence, and significance cutoffs of 0.01, 0.1, and q < 0.05 (Benjamini–Hochberg multiple test correction), respectively^27^ (Suppl. Tables 3 and 4). Taxonomic differences identified via MaAsLin2 were subsequently compared by case type and survival outcome with heat tree visualizations using log2 median ratio differences using metacoder (v0.34)^28^. In order to identify and describe any variability for the observed taxonomic and functional features that was distinctive by case type (i.e., COVID-19, CAP, uninfected) or COVID-19 mortality, we employed Dirichlet multinomial mixture (DMM)^29^ probabilistic modelling. DMM modeling was selected as the means for identifying community clusters due to the algorithm’s ability to generate mixture component vectors based on unique hyperparameters in a multinomial fashion. By design, this methodology intrinsically incorporates dynamic features with ranging sample sizes and species rareity when clustering communities of similar composition, therein making it an optimal tool for this meta-analysis.

Square root scaled GO term counts and taxonomic feature count matrices subjected to unsupervised community typing with DMM clustering (Suppl. Table 5) were subsequently compared by analysis of variance (ANOVA) with metadata categories case type and survival outcome. Statistically significant GO terms results derived from the MaAsLin2 analysis were thereafter ordered by parental lineage and visualized alongside consensus DMM clusters and metadata categories publication, case type, and survival outcome using the package pheatmap (v1.0.12)^30^.

### Data and code availability

Overview of the processing workflow as well as all code used in the execution of the processing pipeline, analysis and visualization R scripts, and intermediate files have been made publicly available can be found online at the COV-IRT microbial GitHub repository (https://github.com/COV-IRT/microbial) and Open Science Framework (OSF) project (https://osf.io/7nrd3/) websites. Additional information about the commands and versions of the tools used to process raw reads and assign taxonomies and GO terms can be found online on the OSF project website (https://osf.io/7nrd3/).

## Results

### Comparison between subject categorical classes (i.e., uninfected controls, or patients with CAP or COVID-19 disease)

**Figure 1:**
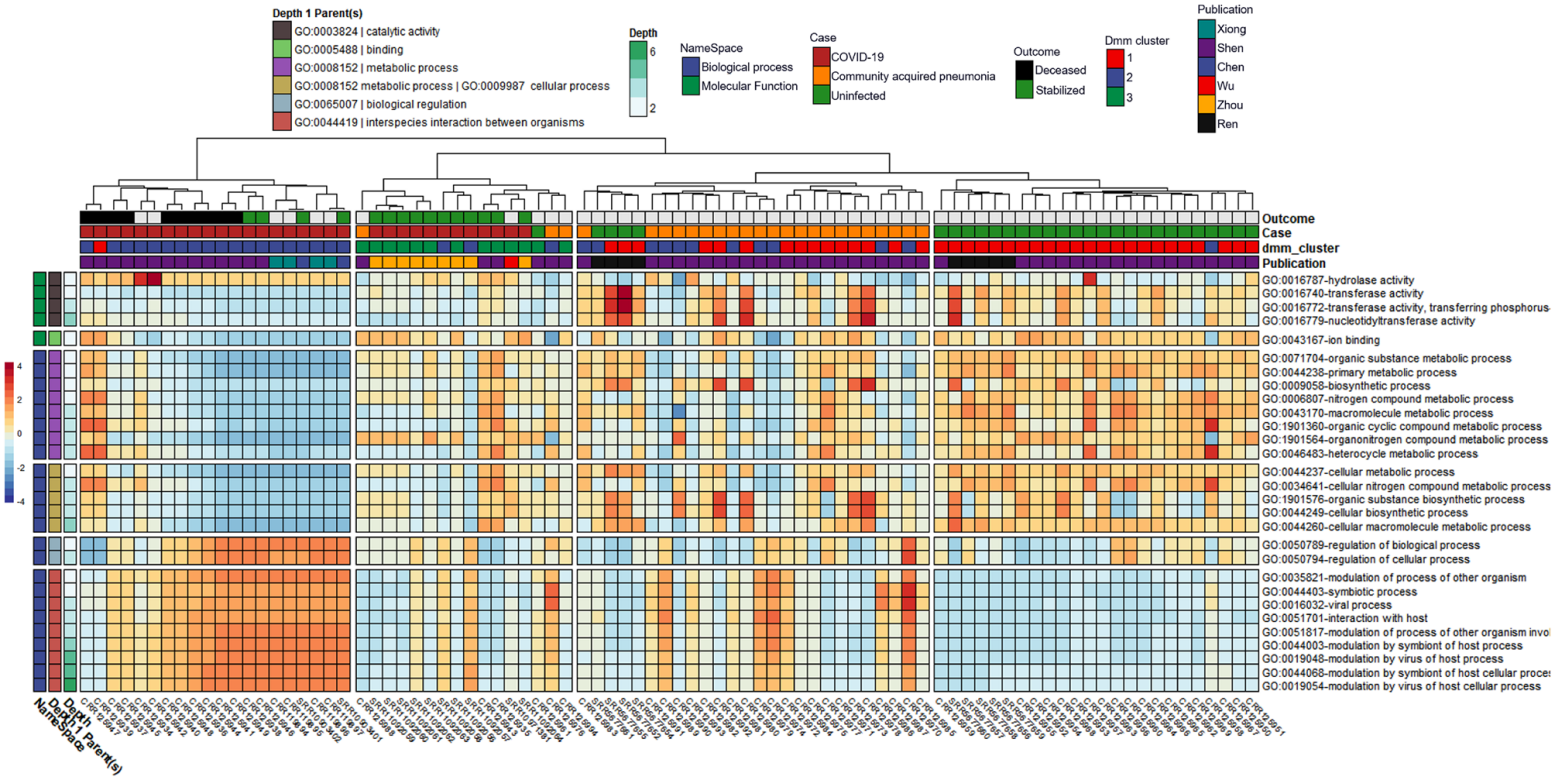
Heatmap with notable microbially-derived gene ontology functional annotations associated with COVID-19 (n =32), as compared to community acquired pneumonia (n = 29) & uninfected (n=25) cohorts. Cells are colored via z-scale calculations of the total read counts for each GO term by sample. Rows are sorted by parental GO terms (depth = 1), and columns are clustered by Euclidean distance using ward D2 clustering. Comparisons were conducted using MaAsLin2, controlling for publication and patient ID with Benjamini Hochberg multiple test comparison (q < 0.05).

After controlling for random effects of publication and patient, results from the MaAsLin2 comparison across individual subjects were grouped by one of three categorical classes: (1) uninfected controls; (2) CAP patients; or (3) COVID-19 patients with moderate to severe disease, including death (Table 1). This revealed 20 out of 13,534 GO terms associated with COVID-19 when compared to the CAP cohort and 30 out of 13,534 GO terms associated with COVID-19 when compared to the uninfected cohort (Fig. 1, Tables 1 and 2). Significant GO terms were grouped under seven parental GO terms, including catalytic activity [GO:0003824], binding [GO.0005488], metabolic process [GO:0008152], cellular process [GO:0009987], biological regulation [GO:0065007], viral process [GO:0016032], and interspecies interaction between organisms [GO:0044419] (Fig. 1, Suppl. Table 3). Parental GO terms have smaller depth numbers (e.g., depth = 1) in the Gene Ontology hierarchy and represent higher-level features under molecular function [GO:0003674] or biological process [GO:0008150], whereas larger depth numbers represent nodes in the ontology tree that are lower and refer to more specific functions or processes.

**Table 2:** MaAsLin2-derived significant gene ontologies associated with COVID-19 (n = 32) when compared to the community acquired pneumonia (n = 25) cohort.

Feature	Namespace	Value (vs COVID-19)	Coef	Stderr	N	Samples w/ > 0 counts	pval	qval
Hydrolase activity	GO:0016787	Community acquired pneumonia	−0.008	0.001	86.000	69.000	0.000	0.000
Cellular process	GO:0009987	Community acquired pneumonia	0.010	0.002	86.000	74.000	0.000	0.000
Transferase activity	GO:0016740	Community acquired pneumonia	0.006	0.002	86.000	58.000	0.000	0.001
Modulation by virus of host cellular process	GO:0019054	Community acquired pneumonia	−0.004	0.001	86.000	11.000	0.000	0.001
Modulation by symbiont of host cellular process	GO:0044068	Community acquired pneumonia	−0.004	0.001	86.000	11.000	0.000	0.001
Biosynthetic process	GO:0009058	Community acquired pneumonia	0.005	0.001	86.000	23.000	0.000	0.001
Cellular macromolecule metabolic process	GO:0044260	Community acquired pneumonia	0.002	0.001	86.000	4.000	0.001	0.003
Organic substance biosynthetic process	GO1901576	Community acquired pneumonia	0.003	0.001	86.000	12.000	0.001	0.004
Cellular biosynthetic process	GO:0044249	Community acquired pneumonia	0.003	0.001	86.000	12.000	0.001	0.004
Transferase activity transferring phosphorus containing groups	GO:0016772	Community acquired pneumonia	0.005	0.001	86.000	14.000	0.001	0.004
Cellular metabolic process	GO:0044237	Community acquired pneumonia	0.004	0.001	86.000	72.000	0.005	0.012
Modulation by virus of host process	GO:0019048	Community acquired pneumonia	−0.004	0.001	86.000	18.000	0.005	0.012
Nucleotidyltransferase activity	GO:0016779	Community acquired pneumonia	0.003	0.001	86.000	7.000	0.006	0.012
Metabolic process	GO:0008152	Community acquired pneumonia	0.005	0.002	86.000	76.000	0.006	0.013
Organonitrogen compound metabolic process	GO:1901564	Community acquired pneumonia	0.002	0.001	86.000	5.000	0.009	0.018
Modulation by symbiont of host process	GO:0044003	Community acquired pneumonia	−0.003	0.001	86.000	18.000	0.018	0.032
Modulation of process of other organism involved in symbiotic interaction	GO:0051817	Community acquired pneumonia	−0.003	0.001	86.000	18.000	0.018	0.032
Modulation of process of other organism	GO:0035821	Community acquired pneumonia	−0.003	0.001	86.000	18.000	0.018	0.032
Organic substance metabolic process	GO:0071704	Community acquired pneumonia	0.003	0.001	86.000	76.000	0.026	0.045
Nucleic acid metabolic process	GO:0090304	Community acquired pneumonia	−0.002	0.001	86.000	17.000	0.029	0.048

Comparisons were conducted using compositional transformed and CLR normalized count matrices, controlled for the random effects of publication and patient, and adjusted for multiple test comparisons using the Benajmini Hochberg correction method. A full list of statistically significant GO terms in COVID-19 vs. CAP can be found in Suppl. Table 3.

**Table 3:** MaAsLin2-derived significant gene ontologies associated with COVID-19 (n = 32) when compared to the uninfected (n = 29) cohort.

Feature	Namespace	Value (vs COVID-19)	Coef	Stderr	N	Samples w/ > 0 counts	pval	qval
Cellular process	GO:0009987	Uninfected	0.016	0.002	86.000	74.000	0.000	0.000
Metabolic process	GO:0008152	Uninfected	0.013	0.002	86.000	76.000	0.000	0.000
Modulation by symbiont of host cellular process	GO:0044068	Uninfected	−0.007	0.001	86.000	11.000	0.000	0.000
Modulation by virus of host cellular process	GO:0019054	Uninfected	−0.007	0.001	86.000	11.000	0.000	0.000
Modulation by virus of host process	GO:0019048	Uninfected	−0.008	0.001	86.000	18.000	0.000	0.000
Organic substance metabolic process	GO:0071704	Uninfected	0.008	0.001	86.000	76.000	0.000	0.000
Cellular macromolecule metabolic process	GO:0044260	Uninfected	0.004	0.001	86.000	4.000	0.000	0.000
Cellular metabolic process	GO:0044237	Uninfected	0.009	0.001	86.000	72.000	0.000	0.000
Modulation by symbiont of host process	GO:0044003	Uninfected	−0.008	0.001	86.000	18.000	0.000	0.000
Modulation of process of other organism	GO:0035821	Uninfected	−0.008	0.001	86.000	18.000	0.000	0.000
Modulation of process of other organism involved in symbiotic interaction	GO:0051817	Uninfected	−0.008	0.001	86.000	18.000	0.000	0.000
Hydrolase activity	GO:0016787	Uninfected	−0.008	0.001	86.000	69.000	0.000	0.000
Interaction with host	GO:0051701	Uninfected	−0.009	0.002	86.000	20.000	0.000	0.000
Viral process	GO:0016032	Uninfected	−0.013	0.002	86.000	31.000	0.000	0.000
Transferase activity	GO:0016740	Uninfected	0.009	0.002	86.000	58.000	0.000	0.000
Primary metabolic process	GO:0044238	Uninfected	0.006	0.001	86.000	74.000	0.000	0.000
Symbiotic process	GO:0044403	Uninfected	−0.014	0.002	86.000	31.000	0.000	0.000
Interspecies interaction between organisms	GO:0044419	Uninfected	−0.014	0.002	86.000	31.000	0.000	0.000
Macromolecule metabolic process	GO:0043170	Uninfected	0.004	0.001	86.000	66.000	0.000	0.000
Organonitrogen compound metabolic process	GO:1901564	Uninfected	0.004	0.001	86.000	5.000	0.000	0.000
Binding	GO:0005488	Uninfected	0.004	0.001	86.000	81.000	0.000	0.001
Nitrogen compound metabolic process	GO:0006807	Uninfected	0.004	0.001	86.000	70.000	0.000	0.001
Biosynthetic process	GO:0009058	Uninfected	0.004	0.001	86.000	23.000	0.005	0.012
Ion binding	GO:0043167	Uninfected	0.002	0.001	86.000	8.000	0.006	0.012
Regulation of biological process	GO:0050789	Uninfected	−0.003	0.001	86.000	15.000	0.010	0.020
Cellular nitrogen compound metabolic process	GO:0034641	Uninfected	0.002	0.001	86.000	53.000	0.011	0.021
Transferase activity transferring phosphorus containing groups	GO:0016772	Uninfected	0.004	0.001	86.000	14.000	0.014	0.027
Catalytic activity	GO:0003824	Uninfected	0.009	0.004	86.000	86.000	0.023	0.041
RNA metabolic process	GO:0016070	Uninfected	0.002	0.001	86.000	6.000	0.028	0.048
Regulation of cellular process	GO:0050794	Uninfected	−0.002	0.001	86.000	12.000	0.030	0.050

Comparisons were conducted using compositional transformed and CLR normalized count matrices, controlled for the random effects of publication and patient, and adjusted for multiple test comparisons using the Benjamini Hochberg correction method. A full list of statistically significant GO terms in COVID-19 vs. Uninfected can be found in Suppl. Table 3.

GO terms enriched in the COVID-19 cohort compared to the uninfected cohort included hydrolase activity [GO:0016787], as well as all significant GO terms with the parental terms of biological regulation [GO:0065007], viral process [GO:0016032], and interspecies interaction between organisms [GO:0044419]. Hydrolase activity [GO:0016787], nucleic acid metabolic process [GO:0090304], and many GO terms classified under interspecies interaction between organisms [GO:0044419] were also enriched in the COVID-19 cohort when compared to CAP. In contrast, GO terms enriched in the uninfected cohort compared to the COVID-19 cohort included all significant GO terms with the parental terms of cellular process [GO:0009987], metabolic process [GO:0008152], binding [GO.0005488], and terms classified under catalytic activity [GO:0003824] other than hydrolase activity [GO:0016787]. Results from the Dirichlet multinomial mixtures clustering analysis using all 13,534 gene ontology counts resulted in a best model fit using three distinct clusters that were significantly associated with each case cohort [p < 0.0001] (Fig. 1, Suppl. Table 5).

Taxonomic comparisons of the COVID-19 cohort to uninfected and CAP cohorts revealed 233 and 61 significantly differentiated species-level taxa with absolute values of log2 median ratios > 1.0 when comparing the COVID-19 cohort to uninfected and CAP cohorts, respectively (Suppl. Table 6). All significant taxa found in the CAP cohort were depleted compared to the COVID-19 cohort. Additionally, all significant taxa found in the CAP to COVID-19 comparison were also identified as significant in the uninfected to COVID-19 comparison (Suppl. Table 6). Of the taxa identified when comparing the uninfected cohort to the COVID-19 cohort, a total of 36 species were only marginally enriched (Suppl. Table 6).

**Figure 2:**
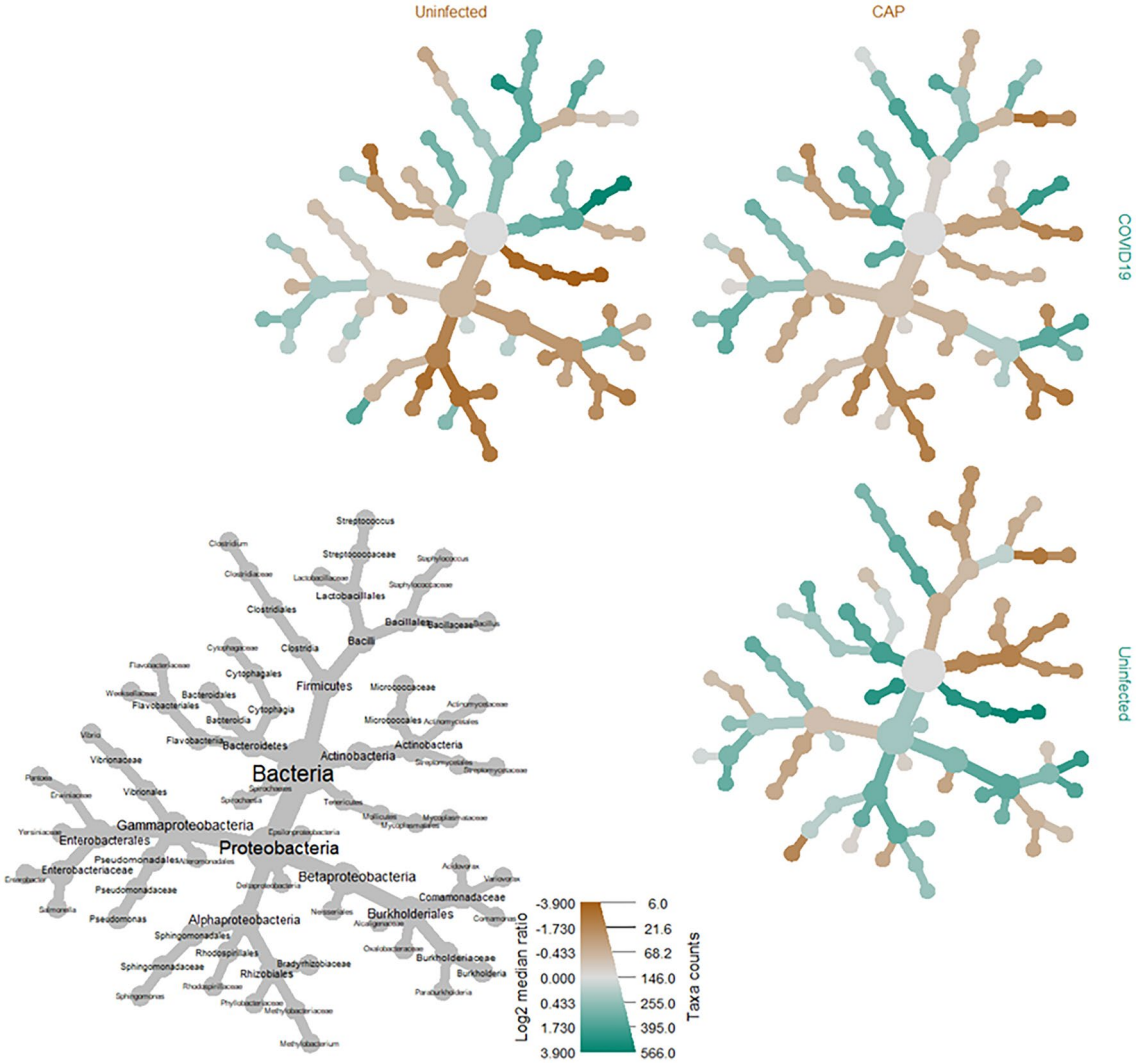
Heat tree matrix visualizing distinct COVID-19 vs. uninfected & community acquired viral pneumonia taxonomic profiles. This taxonomic heat tree data matrix visualization depicts the log2 median ratio differences across the three different cohorts. The tree depicted in grey in the lower left represents a taxonomic assignment key for all the other trees. Each of smaller trees represents a comparison between the different cohorts, as labelled in the columns and rows. The taxa colored brown are more abundant among the cohort labelled in the columns, whereas taxa colored green are more abundant in the cohort labelled in the rows. For example, there were significant increases (green) identified in log2 median ratio of several species belonging to the genus *Sphingomonas* when comparing the COVID-19 to the uninfected cohort (top left) and decreases (brown) when comparing the uninfected cohort to the community acquired pneumonia (CAP) cohort (bottom right).

Taxonomic comparisons resulted in a statistically significant difference amongst several microbial genera within the phylum of Proteobacteria, including those of the families *Sphingomonadaceae* (i.e., *Sphingobium*, *Sphingopyxis*, *Sphingomonas*) and *Rhodobacteraceae* (i.e., *Paracoccus*) when comparing the COVID-19 cohort to the uninfected (p < 0.001, q < 0.001) and CAP (p = 0.0067, q = 0.024) cohorts (Fig. 2, Table 4). There was a significant increase in several species belonging to the genus *Sphingomonas* among BALF specimens from COVID-19 patients when compared to both the uninfected (p < 0.0001, q < 0.001) and CAP cohorts (p < 0.005, q < 0.05) (Suppl. Table 6), with a more significant increase of *Sphingomonas* in COVID-19 patients when compared to the uninfected cohort than to the CAP cohort (Fig. 2). An analysis of the most common SeqScreen outputs taxonomically classified as *Sphingomonas* in BALF specimens among patients with COVID-19, irrespective of disease outcomes, included GO term assignments of hydrogen peroxide catabolic process [GO:0042744], response to oxidative stress [GO:0006979], catalase activity [GO:0004096], heme binding [GO:0020037], and metal ion binding [GO:0046872].

**Table 4:** Log2 median ratio values of top taxa associated with COVID-19 (n = 29) compared to community acquired pneumonia (n = 25) and uninfected (n = 32) cohorts.

Treatment 1	Treatment 2	log2 median ratio	Median diff	Mean diff	p value	q value	Taxon name
COVID-19	Community acquired pneumonia	−2.0539	−0.0728	−0.1332	0.0067	0.0224	*Paracoccus*
COVID-19	Community acquired pneumonia	−2.0539	−0.0728	−0.1332	0.0067	0.0224	*Sphingobium*
COVID-19	Community acquired pneumonia	−2.0539	−0.0728	−0.1332	0.0067	0.0224	*Sphingopyxis*
COVID-19	Community acquired pneumonia	−2.0539	−0.0728	−0.1332	0.0067	0.0224	*Sphingomonas*
COVID-19	Uninfected	−4.3829	−0.4587	−0.3767	< 0.001	< 0.0001	*Paracoccus*
COVID-19	Uninfected	−4.3829	−0.4587	−0.3767	< 0.001	< 0.0001	*Sphingobium*
COVID-19	Uninfected	−4.3829	−0.4587	−0.3767	< 0.001	< 0.0001	*Sphingopyxis*
COVID-19	Uninfected	−4.3829	−0.4587	−0.3767	< 0.001	< 0.0001	*Sphingomonas*
COVID-19	Uninfected	−5.1294	−0.4164	−0.3356	< 0.001	< 0.0001	*Bradyrhizobium*
COVID-19	Uninfected	−5.1294	−0.4164	−0.3356	< 0.001	< 0.0001	*Methylobacterium*

Values depicted in the table are mean values at the genus level for taxa that containing > 10 species level significant comparisons with a qvalue < 0.05 and an absolute value of log2 median ratio > 1.0 using Wilcoxon rank sum test adjusted for multiple test comparison. A full list of significant taxonomic comparison values at the species level can be found in Supplementary Table 6.

There were no significant differences in alpha diversity when comparing case type (i.e., COVID-19, CAP, uninfected) (p-value = 0.051) or mortality (p-value = 0.8918) using the Shannon and inverse Simpson indices^31,32^. A full list of diversity metric indices is available in Supplementary Table 7. Beta diversity analyses did not reveal any statistically significant within group differences (F = 0.293, p = 0.747) by cohorts, which were determined by analysis of variance homogeneity of multivariate dispersions based on Euclidean distance. Further, no significant differences were observed in beta diversity amongst case type (F = 2.9257, p > 0.05) or mortality (F = 3.5978, p > 0.05), as determined by the permutation test for adonis using bray Curtis dissimilarity indices after stratifying by publication and patient.

### Metatranscriptomic comparison of BALF specimens from COVID-19 subjects sub-categorized and stratified by disease survival or death

**Figure 3:**
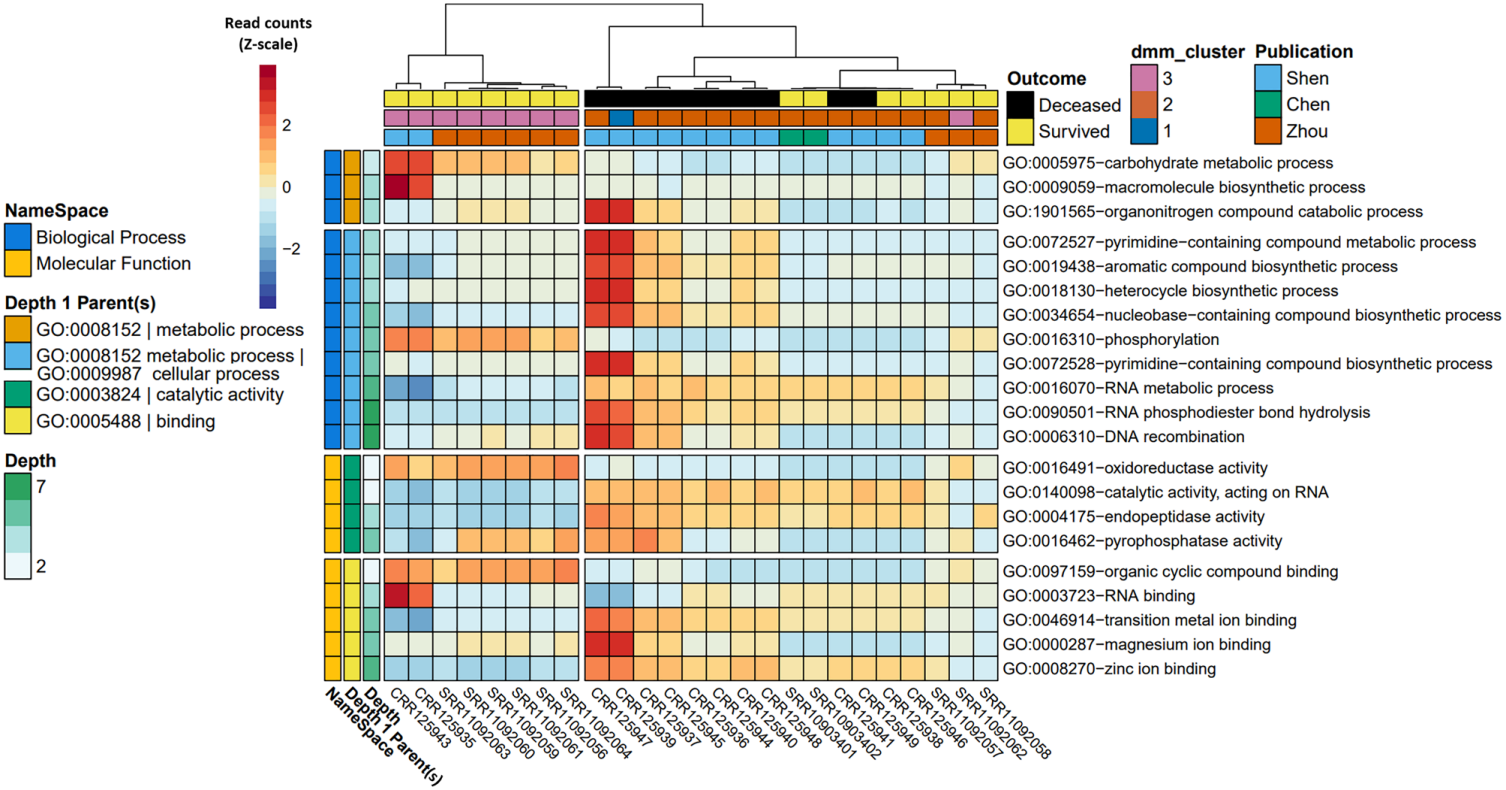
Heatmap of significantly different gene ontology terms associated with COVID-19 mortality comparing deceased (n = 10) versus survived (n = 15). Cells are colored via z-scale calculations of the total read counts by sample (x axis) and by GO term (y axis). Rows are sorted by parental GO terms (depth = 1) and columns are clustered by Euclidean distance using ward D2 clustering. Comparisons were conducted using MaAsLin2, controlling for patient ID with Benjamini Hochberg multiple test comparison (q < 0.05).

From subjects with known COVID-19 survival outcomes (i.e., of 32 samples, *n* = 10 deceased, and *n* = 15 survived), a stratified analysis amongst the categorical class of COVID-19 disease was performed via MaAsLin2. After controlling for random effects of patient, we observed 21 unique GO terms which were significantly increased in their association with death (*n* terms = 16, q-value < 0.05) or survival (*n* terms = 5, q-value < 0.05) from COVID-19 disease, with parental GO terms (depth = 1) of metabolic process [GO:0090304], binding [GO.0005488], and catalytic activity [GO:0003824] (Table 5, Fig. 3). GO terms with significant q-values (< 0.05) that were terminal in the observed GO term lineage (i.e., as specific as possible within the lineages of our result set), included nucleobase-containing compound biosynthetic process [GO:0034654], organonitrogen compound catabolic process [GO:1901565], pyrimidine-containing compound biosynthetic process [GO:0072528], and DNA recombination [GO:0006310] classified under the parental GO term of metabolic process [GO:0008152]; RNA binding [GO:0003723], magnesium ion binding [GO:0000287], and zinc ion binding [GO:0008270] classified under the parental GO term of binding [GO.0005488]; and oxidoreductase activity [GO:0016491] and endopeptidase activity [GO:0004175] classified under the parental GO term of catalytic activity [GO:0003824] (Suppl. Tables 4, 9–17).

**Table 5:** MaAsLin2-derived significant gene ontologies associated with COVID-19 disease outcome (deceased vs. survived).

Name	Ontology	Namespace	Depth	Coef	Stderr	pval	qval	N	Samples w/ > 0 counts
Pyrimidine-containing compound metabolic process	Biological_process	GO:0072527	4	−4.815	0.867	< 0.001	< 0.001	25	12
Nucleobase-containing compound biosynthetic process	Biological_process	GO:0034654	5	−0.630	0.117	< 0.001	< 0.001	25	25
Transition metal ion binding	Molecular_function	GO:0046914	5	−0.545	0.106	< 0.001	< 0.001	25	25
Aromatic compound biosynthetic process	Biological_process	GO:0019438	4	−0.478	0.116	< 0.001	0.004	25	25
Heterocycle biosynthetic process	Biological_process	GO:0018130	4	−0.393	0.100	< 0.001	0.007	25	25
Macromolecule biosynthetic process	Biological_process	GO:0009059	4	0.382	0.103	< 0.001	0.015	25	25
RNA metabolic process	Biological_process	GO:0016070	6	−0.310	0.086	< 0.001	0.018	25	25
RNA phosphodiester bond hydrolysis	Biological_process	GO:0090501	7	−1.412	0.402	< 0.001	0.024	25	17
Magnesium ion binding	Molecular_function	GO:0000287	5	−2.336	0.709	0.001	0.036	25	11
RNA binding	Molecular_function	GO:0003723	4	0.989	0.303	0.001	0.036	25	23
Zinc ion binding	Molecular_function	GO:0008270	6	−0.880	0.266	0.001	0.036	25	24
Phosphorylation	Biological_process	GO:0016310	5	2.897	0.888	0.001	0.036	25	13
Organonitrogen compound catabolic process	Biological_process	GO:1901565	4	−2.388	0.721	0.001	0.036	25	12
Endopeptidase activity	Molecular_function	GO:0004175	4	−0.995	0.309	0.001	0.037	25	21
Pyrimidine-containing compound biosynthetic process	Biological_process	GO:0072528	5	−5.505	1.711	0.001	0.037	25	7
DNA recombination	Biological_process	GO:0006310	7	−2.130	0.667	0.001	0.037	25	12
oxidoreductase activity	Molecular_function	GO:0016491	2	2.541	0.801	0.002	0.037	25	13
Carbohydrate metabolic process	Biological_process	GO:0005975	3	2.245	0.717	0.002	0.039	25	15
Catalytic activity, acting on RNA	Molecular_function	GO:0140098	2	−0.546	0.174	0.002	0.039	25	25
Pyrophosphatase activity	Molecular_function	GO:0016462	5	−0.326	0.107	0.002	0.048	25	25
Organic cyclic compound binding	Molecular_function	GO:0097159	2	0.443	0.145	0.002	0.048	25	25
Hydrolase activity, acting on acid anhydrides	Molecular_function	GO:0016817	3	−0.323	0.107	0.003	0.052	25	25

Comparisons were conducted using compositional transformed and CLR normalized count matrices, controlled for the random effect of patient ID, and adjusted for multiple test comparisons using the Benajmini Hochberg correction method. A full list of statistically significant GO terms in COVID-19 Deceased vs. Survived can be found in Suppl. Table 4.

**Figure 4:**
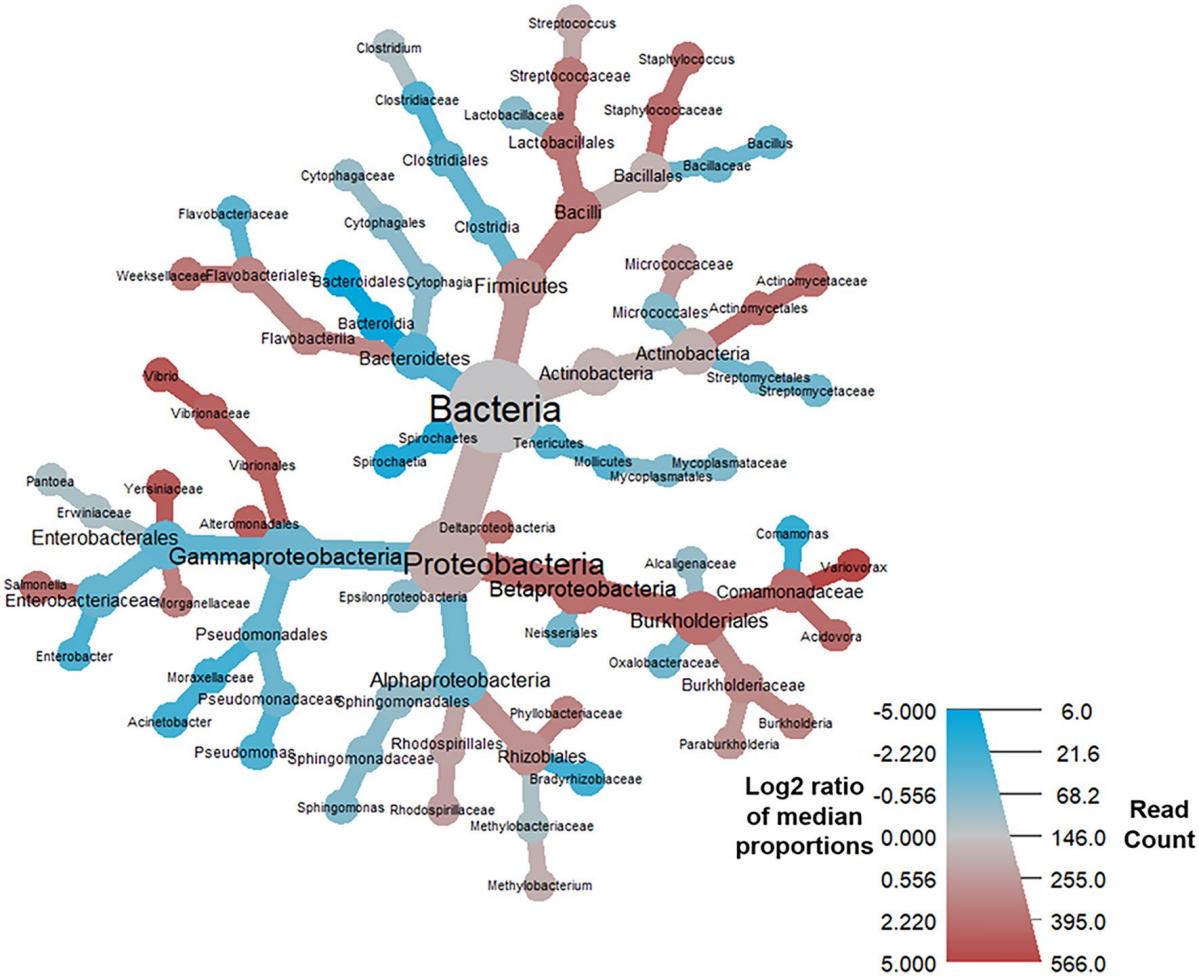
Heat tree demonstrating the BALF metatransciptome profiles associated with COVID-19 mortality. Taxa colored in red were more prevalent amongst COVID-19 patients who died, and nodes in blue represent taxa that were more prevalent amongst patients who survived COVID-19. Notable increases were observed in the log2 median ratios in the family Comamonadaceae, genus *Variovorax,* and significant decreases in the log2 median ratios of order Bacteroidia and class Bacteroidales*.*

Of the nine terminal GO terms that were significantly different in this analysis (q-value < 0.05), RNA binding [GO:0003723] and oxidoreductase activity [GO:0016491] were the most enriched in samples from individuals that survived COVID-19 (Suppl. Table 4). An analysis of the proteins underlying the SeqScreen GO term assignments showed that RNA binding [GO:0003723] was driven by an enrichment of 30S and 50S ribosomal proteins from the Gram-positive cocci belonging to the genera *Streptococcus*, *Granulicatella*, *Enterococcus*, and *Lactococcus*, all of which were particularly prevalent in the “nCov7” survived COVID-19 patient from the Shen et al*.* study (Suppl. Table 8). The enrichment of the oxidoreductase activity [GO:0016491] term among survived COVID-19 patients was driven by many different samples and a variety of bacteria, including those from Gram-positive bacteria belonging to the genera *Enterococcus*, *Streptococcus*, *Streptomyces*, *Pediococcus*, *Lactococcus*, and *Granulicatella*. Examples of underlying reference proteins to which reads mapped resulting in our observed oxidoreductase activity [GO:0016491] term included quinone oxidoreductase, pyruvate dehydrogenase, glyceraldehyde-3-phosphate dehydrogenase, and glyceraldehyde-3-phosphate dehydrogenase (Suppl. Table 14). Among the deceased COVID-19 patients, the terminal GO terms of endopeptidase activity [GO:0004175], zinc ion binding [GO:0008270], and nucleobase-containing compound biosynthetic process [GO:0034654] were being driven by an enrichment of SARS-CoV-2 proteins (Suppl. Tables 10, 12, 14). Mixed among proteins from other organisms, an enrichment of *Variovorax* proteins tagged with the terminal GO terms of pyrimidine-containing compound biosynthetic process [GO:0072528] (e.g., CTP synthase, putative sulfonate/nitrate transport system substrate-binding protein), organonitrogen compound catabolic process [GO:1901565] (e.g., histidine ammonia-lyase, aspartate/glutamate leucyltransferase), magnesium ion binding [GO:0000287] (e.g., proteins involved in the histidine biosynthesis pathway, such as phosphoribosyl-AMP cyclohydrolase), and DNA recombination [GO:0006310] (e.g., inclusive of possible *Variovorax* phage proteins—integrase family protein, putative transposase IS4 family, phage integrase family protein) appeared in the COVID-19 deceased patients. This enrichment of *Variovorax* proteins among samples from individuals who died of COVID-19 was consistent with the results from the taxonomic comparison analysis. Compared to the survived group, the taxonomic comparisons in the deceased group revealed a statistically significant (p < 0.0001, q < 0.001) increase of the family Comamonadaceae, belonging to the genus *Variovorax*, and decreases in the family Bacteriodales (Fig. 4, Table 6).

**Table 6:** Log2 median ratio counts of taxa associated with COVID-19 mortality when comparing deceased (n = 10) versus survived (n = 15).

log_2_ median ratio	Median diff	Mean diff	p value	q value	Taxonomy
2.25	0.361	0.371	0.00017	0.00691	Comamonadaceae
5.21	0.405	0.377	0.00017	0.00691	*Variovorax*
2.97	0.002	0.002	0.00353	0.074	Vibrionales
2.97	0.002	0.002	0.00353	0.074	Vibrionaceae
3.8	0.002	0.00181	0.00492	0.0827	*Vibrio*
1.84	0.0549	0.13	0.0137	0.124	*Bacilli*
2.24	0.403	0.297	0.0163	0.124	Burkholderiales
3.16	0.002	0.002	0.0157	0.124	Alteromonadales
3.61	0.004	0.004	0.0156	0.124	Yersiniaceae
2.1	0.005	0.00435	0.0156	0.124	*Salmonella*
1.77	0.011	0.064	0.0475	0.274	Streptococcaceae
2.29	0.425	0.296	0.0264	0.185	Betaproteobacteria
−5.13	−0.103	−0.104	0.0308	0.199	Bacteroidia
−5.18	−0.099	−0.102	0.00962	0.124	Bacteroidales

Comparisons were conducted using Wilcoxon rank sum test and adjusted for multiple test comparisons using the Benjamini Hochberg correction method. A full list of significant taxonomic comparison values at the species level can be found in Supplementary Table 8.

## Discussion

We observed significantly unique discriminant taxonomic and functional features in bronchoalveolar lavage fluid (BALF) metatranscriptomes in association with COVID-19 disease and its mortality. Of note, due to limitations in the depth of clinical metadata by subject, we could not distinguish between COVID-19 pathophysiology or associated medical comorbidities, treatments, nor interventions. However, because of the time interval in which COVID-19 patient specimens were recruited to their respective index studies at the beginning of the outbreak in Wuhan, China (i.e., 2019 and early 2020), COVID-19-specific interventions and treatments had yet to be introduced and thus comparisons between CAP and COVID-19 subject specimens would be less likely to be related to disease-focused therapy.

### Results driven by coronavirus protein functions

At the time of this study, the standard Kraken2 taxonomic database included the SARS-CoV-2 reference genome, but the SARS-CoV-2 proteins were not yet added to the SeqScreen database that was used for the functional analysis. This functional analysis demonstrated how GO terms and their corresponding proteins can be used to characterize an emerging pathogen (i.e., a pathogen that is not present in the reference database), as well as significant host microbiome functional shifts. SARS-CoV-2 reads were successfully detected in the taxonomic analysis of COVID-19 BALF samples, and GO terms associated with coronavirus proteins were found to be significantly different in the functional analysis. A number of coronavirus proteins were driving the significant associations of GO terms between COVID-19 and uninfected samples, including modulation by symbiont of host cellular process [GO:0044068], modulation by virus of host cellular process [GO:0019054], modulation by virus of host process [GO:0019048], modulation of process of other organism involved in symbiotic interaction [GO:0051817], modulation by symbiont of host process [GO:0044003], interaction with host [GO:0016032], viral process [GO:0051701], interspecies interaction between organisms [GO:0044419], modulation by symbiont of host cellular process [GO:0044068], and modulation by virus of host cellular process [GO:0019054] (Suppl Table 3). Coronavirus proteins were also driving notable GO term associations in COVID-19 deceased vs. survived, including transition metal ion binding [GO:0046914], zinc ion binding [GO:0008270], organic cyclic compound binding [GO:0097159], endopeptidase activity [GO:0004175], and nucleobase containing compound biosynthetic process [GO:0034654]. While samples from both COVID-19 deceased and survived individuals contained taxonomically and functionally classified coronavirus reads, the significant terminal GO terms of endopeptidase activity [GO:0004175], zinc ion binding [GO:0008270], and nucleobase-containing compound biosynthetic process [GO:0034654] were positively correlated with COVID-19 deceased patients. This was likely due to multiple highly expressed coronavirus proteins being tagged with these GO terms (e.g., replicase polyprotein 1ab, 2'-O-methyltransferase), and a higher SARS-CoV-2 viral load and mRNA expression being present in patients who died of COVID-19 disease.

### Significant taxonomic differences observed in the microbial communities

Distinct taxonomic features of BALF specimens from the COVID-19 vs. uninfected analysis included an increase in the genus *Sphingomonas,* belonging to the Sphingomonadacae family, among COVID-19 patients. Notable taxonomic features among COVID-19 patients with mortal disease included increases in log_2_ median ratios of the genus *Variovorax,* belonging to the Comamonadaceae family, and decreases in the class Bacteroidia*,* belonging to the order Bacteroidales. These findings support previous reports regarding an association with Gram-negative *Sphingomonas*^33–36^, which is a common opportunistic pathogen found in nosocomial infections. A previous 16S rRNA profiling study by Gaibani et al. found that the BALF of critically ill COVID-19 patients had lower amounts of commensal bacterial species and an enrichment of opportunistic Gram-negative pathogens, which was often associated with multidrug resistance^40^. Among the COVID-19 cohort, one of the most highly expressed *Sphingomonas* genes was catalase [UniProt ID = J8VPL9]. This *Sphingomonas* catalase protein is assigned GO terms including hydrogen peroxide catabolic process [GO:0042744], response to oxidative stress [GO:0006979], catalase activity [GO:0004096], heme binding [GO:0020037], and metal ion binding [GO:0046872], and it is responsible for decomposing hydrogen peroxide into water and oxygen. This serves to protect cells from the toxic effects of hydrogen peroxide, which may suggest that *Sphingomonas* spp. respond to COVID-19 conditions in the patient by expressing genes that help them to survive well in environments undergoing great amounts of oxidative stress.

Our findings additionally support a previous report regarding an increase in the abundance of *Variovorax* in COVID-19 patient BALF tissue^37^. *Variovorax* spp. Have also previously been reported in the microbiota of patients with lung cancer^38^ and were shown to be a key driver of clustering amongst patients challenged with H1N1 influenza infections^39^. The most abundantly expressed *Variovorax* proteins in the COVID-19 cohort included those involved in cell wall organization and the plasma membrane (e.g., binding-protein-dependent transport systems inner membrane component [UniProt ID = E6VB76], endolytic peptidoglycan transglycosylase RlpA [UniProt ID = T1XG48]), oxidoreductase activity (e.g., methylenetetrahydrofolate reductase [UniProt IDs = J2L4W7, T1XH55], taurine dioxygenase [UniProt ID = T1XBI4], NADH-quinone oxidoreductase subunit H [UniProt ID = E6V509]), hydrolase activity (e.g., *N*-acyl-d-aspartate/d-glutamate deacylase [UniProt ID = J2T0U3], cytokinin riboside 5'-monophosphate phosphoribohydrolase [UniProt IDs = E6V0P4, J3CLH3]), and ATP-binding transport (e.g., ABC transporter related protein [UniProt ID = E6UUY9], extracellular solute-binding protein family 5 [UniProt ID = E6V3F7]).

These findings of this study are consistent with a prior 16S rRNA profiling study by Bassis et al., where BALF from healthy subjects was found to contain bacteria from the genera Prevotella (class Bacteroidia), Veillonella, and Streptococcus^41^. In addition to the significance of the RNA binding GO term [GO:0003723] being driven by an enrichment of 30S and 50S ribosomal proteins from Gram-positive cocci like *Streptococcus* in the survived COVID-19 cohort (Suppl. Table 8), the endopeptidase activity GO term [GO:0004175] was connected to membrane organization proteins for Gram-positive bacteria (e.g., Gram-positive signal peptide protein, YSIRK family), which were more prevalent in the COVID-19 survived cohort. This study also found the class Bacteroidia to be increased in the survived COVID-19 cohort.

### Enrichment of the histidine biosynthesis pathway

The genes underlying the significant GO term magnesium ion binding [GO:0000287] revealed an enrichment of transcripts involved in microbial biosynthesis pathways in the COVID-19 deceased cohort (e.g., Phosphoribosyl-AMP cyclohydrolase)*.* Prior experiments have found that histidine biosynthesis is critical for the pathogen *Klebsiella* *pneumoniae* to grow in immunosuppressed lungs^42^, and histidine serves as a crucial nitrogen source for infections by the nosocomial pathogen *Acinetobacter* *baumannii*^43^. For these reasons, it was proposed that the histidine biosynthesis pathway could be a promising drug target to combat opportunistic bacterial infections^42,43^. In this study, the enrichment of gene transcripts within the histidine biosynthesis pathway among the COVID-19 deceased cohort suggests that histidine could be an important contributor to the survival and pathogenicity of opportunistic bacteria in the BALF of COVID-19 patients.

### Evidence of stress responses in bacterial pathogens

Several of the enriched gene transcripts identified in this study were involved in different bacterial stress response pathways. The zinc ion binding GO term [GO:0008270] was enriched in SARS-CoV-2 proteins, but it was also connected to an increased expression of genes involved in the formaldehyde bacterial stress response in COVID-19 deceased individuals (e.g., *S*-(hydroxymethyl)glutathione dehydrogenase, Glutathione-independent formaldehyde dehydrogenase). Formaldehyde is highly toxic to microbes, and this study showed evidence of genes within the most widespread pathway for formaldehyde detoxification^44^ (where thiol in tripeptide glutathione serves as the initial formaldehyde acceptor) to be enriched in the COVID-19 deceased cohort. Also enriched in the COVID-19 deceased cohort were genes labeled with the DNA recombination GO term [GO:0006310] and involved in phage activity (e.g., *Variovorax* phage proteins—integrase family protein, putative transposase IS4 family, phage integrase family protein). Prophage activities have been previously shown to contribute to the survival and pathogenicity of bacteria and may be activated in response to stress^45–48^. The enrichment of the oxidoreductase activity GO term [GO:0016491] among the COVID-19 survived cohort included underlying genes such as quinone oxidoreductase, pyruvate dehydrogenase, glyceraldehyde-3-phosphate dehydrogenase, and glyceraldehyde-3-phosphate dehydrogenase. Lung disease may become more severe in COVID-19 with increased oxidative stress, and it is possible that bacterial response in the COVID-19 survived cohort helped to reduce the oxidative stress^49–51^.

## Conclusions

COVID-19 disease has demonstrated a wide range of clinical severity outcomes, but the factors that correlate with disease severity are not fully understood. Here we identified significant taxonomic and functional differences in BALF metatranscriptomes associated with COVID-19 disease and death. More significant differences were observed between the COVID-19 disease and uninfected cohorts than the COVID-19 disease and CAP cohorts, suggesting correlations specific to SARS-CoV-2 infection. Significant differences were also found associated with COVID-19 mortality. Discriminant taxonomic differences associated with COVID-19 disease and mortality included the following: the genus *Sphingomonas* significantly increased with COVID-19 disease compared to the uninfected cohort and to a lesser extent with COVID-19 disease compared to the CAP cohort, the genus *Variovorax* significantly increased with COVID-19 mortality, and in the class Bacteroidia significantly decreased with COVID-19 mortality. Compared to the patients who were reported to have survived COVID-19 disease, the metatranscriptome data from COVID-19 deceased individuals showed a significant increase in specific GO terms assigned to SARS-CoV-2 proteins, which was likely because of their higher SARS-CoV-2 viral load. Additionally, COVID-19 deceased individuals showed more transcripts from genes involved in the histidine biosynthesis pathway and demonstrated evidence of active bacterial stress response pathways. By the nature of this analysis, this work does not address causality or directionality. However, this work does identify a relationship between the human microbiome and COVID-19 morbidity and mortality, and the specific functions and taxa identified warrant further investigation. Although this experiment was focused on the impact of COVID-19 disease on the BALF microbiome, none of the methods employed in this study were specific to COVID-19. We hope that the methods implemented here will be useful to the research community for other microbiome and pathogen-related association experiments, particularly as more metatranscriptome sequences and pathogenesis gene ontologies are created in the future^52,53^.

## Supplementary Information

Supplementary Tables.

Supplementary Table 3.

Supplementary Table 4.

Supplementary Table 5.

Supplementary Table 6.

Supplementary Table 7.

Supplementary Table 8.

Supplementary Table 9.

Supplementary Table 10.

Supplementary Table 11.

Supplementary Table 12.

Supplementary Table 13.

Supplementary Table 14.

Supplementary Table 15.

Supplementary Table 16.

Supplementary Table 17.

Supplementary Table 18.

Supplementary Legends.

## Data Availability

The original sequence datasets used in this study were previously published and are publicly available in the locations described in Suppl. Tables 1 and 2. An overview of the data processing workflow, all code used in the execution of the processing pipeline, analysis and visualization R scripts, and intermediate files have been made publicly available can be found online at the COV-IRT microbial GitHub repository (https://github.com/COV-IRT/microbial) and Open Science Framework (OSF) project (https://osf.io/7nrd3/; doi: 10.17605/OSF.IO/7NRD3) websites. The OSF wiki (https://osf.io/7nrd3/wiki/home/) describes specific software tools and commands that were used to generate the results. The OSF project includes the following high-level project components relevant to this manuscript: Microbial_Pre-Processing (i.e., outputs from quality trimming and filtering of raw sequence data), Metatranscriptome_Kraken2 (i.e., Kraken2 taxonomic classification outputs), Metatranscriptome_SeqScreen (i.e., SeqScreen final reports), Metatranscriptome_GO_Terms and Metatranscriptome_GO_Term_Summaries (i.e., summaries of SeqScreen-assigned GO terms), and Metatranscriptome_UniProt_ID_Counts (i.e., summaries of SeqScreen-assigned UniProt IDs). All methods were carried out in accordance with relevant guidelines and regulations. Suppl. Table 18 provides legends for all supplementary tables.
