# Supplementary material for: Analysis of bronchoalveolar lavage fluid metatranscriptomes among patients with COVID-19 disease
Source: Sci Rep. 2022 Dec 7;12:21125. doi: 10.1038/s41598-022-25463-0 (PMC9729217; doi:10.1038/s41598-022-25463-0)
Supplement: Supplementary file 1 — Supplementary Tables. [file 41598_2022_25463_MOESM1_ESM.docx]

**Supplemental Table 1. COVID-19 BALF Data Sources**

| **Publication** | **BioProject*** | **COVID-19 Accession Numbers (Sample Name)** | **Additional COVID-19 Microbiome-Related Analyses** |
| --- | --- | --- | --- |
| Chen et al. 2020 [[1]](https://paperpile.com/c/Y03cM1/ujGB) | NCBI BioProject PRJNA601736 | SRR10903401 (patient 2),  SRR10903402 (patient 1) | Yang et al. 2020 [11] |
| Wu et al. 2020 [[2]](https://paperpile.com/c/Y03cM1/m6N1) | NCBI BioProject PRJNA603194 | SRR10971381 (sample 1) | Abouelkhair 2020 [12]; Khan et al. 2020 [13] |
| Zhou et al. 2020a [[3]](https://paperpile.com/c/Y03cM1/fn51) | NCBI BioProject PRJNA605983 | SRR11092058 (WIV02, repeat 1),  SRR11092063 (WIV02, repeat 2),  SRR11092057 (WIV04, repeat 1),  SRR11092062 (WIV04, repeat 2),  SRR11092061 (WIV05),  SRR11092060 (WIV06, repeat 1),  SRR11092056 (WIV06, repeat 2),  SRR11092059 (WIV07, repeat 1),  SRR11092064 (WIV07, repeat 2) | Yang et al. 2020 [11]; Abouelkhair 2020 [12]; Martino et al. 2020 [14] |
| Xiong et al. 2020 [[4]](https://paperpile.com/c/Y03cM1/Dy9O) | NGDB BioProject PRJCA002326 | Peripheral blood mononuclear cell samples: CRR119891 (PBMC P1), CRR119892 (PBMC P2), CRR119893 (PBMC P3)  Bronchoalveolar lavage fluid samples:  CRR119894 (BALF patient 1, repeat 1),  CRR119895 (BALF patient 1, repeat 2),  CRR119896 (BALF patient 2, repeat 1),  CRR119897 (BALF patient 2, repeat 2) | Yang et al. 2020 [11] |
| Shen et al. 2020 [[5]](https://paperpile.com/c/Y03cM1/Yuay) | NCBI BioProject PRJNA605907 | The paired and single-end reads in each accession were analyzed separately:  SRR11059947 (nCov1), SRR11059946 (nCov2), SRR11059945 (nCov3), SRR11059944 (nCov4), SRR11059943 (nCov5), SRR11059942 (nCov6), SRR11059941 (nCov7), SRR11059940 (nCov8) |  |
| Shen et al. 2020 [[5]](https://paperpile.com/c/Y03cM1/Yuay) | NGDB BioProject PRJCA002202 | CRR125949 (nCov1), CRR125941 (nCov1), CRR125948 (nCov2), CRR125940 (nCov2), CRR125947 (nCov3), CRR125939 (nCov3), CRR125946 (nCov4), CRR125938 (nCov4), CRR125945 (nCov5), CRR125937 (nCov5), CRR125944 (nCov6), CRR125936 (nCov6), CRR125943 (nCov7), CRR125935 (nCov7), CRR125942 (nCov8), CRR125934 (nCov8) | Haiminen et al. 2021 [9]; Zhou et al. 2020b [10]; Yang et al. 2020 [11]; Martino et al. 2020 [14]; Branda et al. 2020 [15] |

**Supplemental Table 2. Non-COVID-19 BALF Data Sources**

| **Publication** | **BioProject*** | **Non-COVID-19 Accession Numbers (Sample Name)** | **Additional COVID-19 Microbiome-Related Analyses** |
| --- | --- | --- | --- |
| Xiong et al. 2020 [[4]](https://paperpile.com/c/Y03cM1/Dy9O) | NGDB BioProject PRJCA002326 | Peripheral blood mononuclear cell samples:  CRR119890 (PBMC N1), CRR125445 (PBMC N2), CRR125446 (PBMC N3) |  |
| Shen et al. 2020 [[5]](https://paperpile.com/c/Y03cM1/Yuay) | NGDB BioProject PRJCA002202 | Healthy individuals:  CRR125950 (H01), CRR125951 (H02),  CRR125952 (H03), CRR125953 (H04),  CRR125954 (H05), CRR125955 (H06),  CRR125956 (H07), CRR125957 (H08),  CRR125958 (H09), CRR125959 (H10),  CRR125960 (H11), CRR125961 (H12),  CRR125962 (H13), CRR125963 (H14),  CRR125964 (H15), CRR125965 (H16),  CRR125966 (H17), CRR125967 (H18),  CRR125968 (H19), CRR125969 (H20)  Individuals with community acquired pneumonia, likely viral infections, with sequence identified respiratory virus:  Human mastadenovirus B  CRR125970 (V01)  Measles morbillivirus  CRR125975 (V06)  Human coronavirus HKU1  CRR125986 (V17)  Rhinovirus A  CRR125971 (V02), CRR125973 (V04),  CRR125974 (V05), CRR125981 (V12),  CRR125982 (V13), CRR125990 (V21)  Rhinovirus B  CRR125972 (V03), CRR125988 (V19),  CRR125989 (V20)  Rhinovirus C  CRR125976 (V07)  Influenza A  CRR125977 (V08), CRR125978 (V09),  CRR125979 (V10), CRR125987 (V18)  Influenza B  CRR125980 (V11), CRR125992 (V23)  Enterovirus D  CRR125983 (V14), CRR125984 (V15),  CRR125985 (V16)  Human orthopneumovirus  CRR125991 (V22), CRR125993 (V24),  CRR125994 (V25)  Negative controls:  CRR125995 (nc1), CRR125996 (nc2),  CRR125997 (nc3), CRR125998 (nc3_se) | Haiminen et al. 2021 [[9]](https://paperpile.com/c/Y03cM1/pvat); Zhou et al. 2020b [[10]](https://paperpile.com/c/Y03cM1/3UGH); Yang et al. 2020 [11]; Martino et al. 2020 [14]; Branda et al. 2020 [15] |
| Michalovich et al. 2019 [[6]](https://paperpile.com/c/Y03cM1/0Oiw) | NCBI BioProject PRJNA434133 | Obese, asthma, non-smokers:  SRR10571663 (SIB004), SRR10571660 (SIB006),  SRR10571655 (SIB012),  SRR10571743 (SIB015), SRR10571725 (SIB035), SRR10571723 (SIB037), SRR10571757 (SIB044), SRR10571755 (SIB046), SRR10571752 (SIB049)  Obese, non-asthma, non-smokers:  SRR10571662 (SIB005), SRR10571657 (SIB010), SRR10571744 (SIB014), SRR10571736 (SIB023), SRR10571735 (SIB024),  SRR10571734 (SIB025)  Obese, non-asthma, smokers:  SRR10571664 (SIB003), SRR10571659 (SIB007),  SRR10571658 (SIB008), SRR10571656 (SIB011), SRR10571741 (SIB018)  Obese, asthma, smokers:  SRR10571756 (SIB045)  Non-obese, non-asthma, smokers:  SRR10571728 (SIB031), SRR10571739 (SIB019), SRR10571754 (SIB047)  Non-obese, asthma, non-smokers:  SRR10571665 (SIB002), SRR10571742 (SIB016), SRR10571738 (SIB021), SRR10571737 (SIB022), SRR10571731 (SIB029), SRR10571722 (SIB038), SRR10571720 (SIB042)  Non-obese, asthma, smokers:  SRR10571727 (SIB032), SRR10571719 (SIB043)  Non-obese, asthma, ex-smokers:  SRR10571726 (SIB033), SRR10571721 (SIB039), SRR10571753 (SIB048)  Non-obese, non-asthma, non-smokers:  SRR10571732 (SIB028), SRR10571730 (SIB030), SRR10571724 (SIB036) | Xiong et al. 2020 [[4]](https://paperpile.com/c/Y03cM1/Dy9O); Yang et al. 2020 [11] |
| Huang et al. 2019 [[7]](https://paperpile.com/c/Y03cM1/Cm28) | NCBI BioProject PRJNA484025 | SRR7788525+SRR7638291 (S01),  SRR7788526+SRR7638290 (S02),  SRR7788523+SRR7638293 (S03),  SRR7788524+SRR7638289 (S04),  SRR7788529+SRR7638295 (S05),  SRR7788530+SRR7638294 (S06),  SRR7788527+SRR7638297 (S07),  SRR7788528+SRR7638296 (S08),  SRR7788532+SRR7638288 (S09),  SRR7788533+SRR7638287 (S10),  SRR7788515+SRR7638280 (S11),  SRR7788516+SRR7638279 (S12),  SSRR7788513+SRR7638278 (S13),  SSRR7788514+SRR7638277 (S14),  SRR7788519+SRR7638284 (S15),  SRR7788520+SRR7638283 (S16),  SRR7788517+SRR7638282 (S17),  SRR7788518+SRR7638281 (S18),  SRR7788521+SRR7638286 (S19),  SRR7788522+SRR7638285 (S21),  SRR7788531+SRR7638292 (S22)  Negative control:  SRR7796663 (NC1) |  |
| Ren et al 2018 [[8]](https://paperpile.com/c/Y03cM1/bZke) | NCBI BioProject PRJNA390194 | SRR5677656 (ANB21),  SRR5677657 (ANB22),  SRR5677654 (ANB23),  SRR5677655 (ANB24),  SRR5677660 (ANB26),  SRR5677661 (ANB30),  SRR5677658 (ANB32),  SRR5677659 (ANB33),  SRR5677652 (ANB40) |  |

*NCBI BioProject accession numbers begin with SRR (<https://www.ncbi.nlm.nih.gov/bioproject/>) and NGDC BioProject accession numbers begin with CRR (<https://bigd.big.ac.cn/bioproject/>)

**References**

1. Chen L, Liu W, Zhang Q, Xu K, Ye G, Wu W, et al. RNA based mNGS approach identifies a novel human coronavirus from two individual pneumonia cases in 2019 Wuhan outbreak. Emerg Microbes Infect. 2020;9: 313–319. doi:10.1080/22221751.2020.1725399

2. Wu F, Zhao S, Yu B, Chen Y-M, Wang W, Song Z-G, et al. A new coronavirus associated with human respiratory disease in China. Nature. 2020;579: 265–269. doi:10.1038/s41586-020-2008-3

3. Zhou P, Yang X-L, Wang X-G, Hu B, Zhang L, Zhang W, et al. A pneumonia outbreak associated with a new coronavirus of probable bat origin. Nature. 2020;579: 270–273. doi:10.1038/s41586-020-2012-7

4. Xiong Y, Liu Y, Cao L, Wang D, Guo M, Jiang A, et al. Transcriptomic characteristics of bronchoalveolar lavage fluid and peripheral blood mononuclear cells in COVID-19 patients. Emerg Microbes Infect. 2020;9: 761–770. doi:10.1080/22221751.2020.1747363

5. Shen Z, Xiao Y, Kang L, Ma W, Shi L, Zhang L, et al. Genomic diversity of severe acute respiratory syndrome–coronavirus 2 in patients with coronavirus disease 2019. Clinical Infectious Diseases. 2020;71(15): 713-720. doi:10.1093/cid/ciaa203

6. Michalovich D, Rodriguez-Perez N, Smolinska S, Pirozynski M, Mayhew D, Uddin S, et al. Obesity and disease severity magnify disturbed microbiome-immune interactions in asthma patients. Nat Commun. 2019;10: 5711. doi:10.1038/s41467-019-13751-9

7. Huang W, Yin C, Wang G, Rosenblum J, Krishnan S, Dimitrova N, et al. Optimizing a metatranscriptomic next-generation sequencing protocol for bronchoalveolar lavage diagnostics. J Mol Diagn. 2019;21: 251–261. doi:10.1016/j.jmoldx.2018.09.004

8. Ren L, Zhang R, Rao J, Xiao Y, Zhang Z, Yang B, et al. Transcriptionally active lung microbiome and Its association with bacterial biomass and host inflammatory status. mSystems. 2018;3. doi:[10.1128/mSystems.00199-18](http://dx.doi.org/10.1128/mSystems.00199-18)

9. Haiminen N, Utro F, Seabolt E, Parida L. Functional profiling of COVID-19 respiratory tract microbiomes. Sci Rep. 2021;11(1):6433. doi:10.1038/s41598-021-85750-0

10. Zhou Z, Ren L, Zhang L, Zhong J, Xiao Y, Jia Z, et al. Heightened innate immune responses in the respiratory tract of COVID-19 patients. Cell Host Microbe. 2020;27: 883–890.e2. doi:10.1016/j.chom.2020.04.017

11. Yang H, Zhilong J, Jinlong S, Weidong W, Kunlun H. The active lung microbiota landscape of COVID-19 patients through the metatranscriptome data analysis. BioImpacts. 2021; BI 12 (2): 139–46. doi:10.34172/bi.2021.23378

12. Abouelkhair MA. Non-SARS-CoV-2 genome sequences identified in clinical samples from COVID-19 infected patients: Evidence for co-infections. PeerJ. 2020;8:e10246. doi:10.7717/peerj.10246

13. Khan AA, Khan Z. COVID-2019-associated overexpressed Prevotella proteins mediated host-pathogen interactions and their role in coronavirus outbreak. Bioinformatics. 2020;36(13):4065-4069. doi:10.1093/bioinformatics/btaa285

14. Martino C, Kellman BP, Sandoval DR, et al. Bacterial modification of the host glycosaminoglycan heparan sulfate modulates SARS-CoV-2 infectivity. Preprint. bioRxiv. 2020;2020.08.17.238444. Published 2020 Aug 18. doi:10.1101/2020.08.17.238444

15. Branda S, Poorey K. COVID-19 biomarkers based on respiratory microbiome content. United States: N. p., 2020. Web. doi:10.2172/1673815
