# Supplementary material for: Analysis of bronchoalveolar lavage fluid metatranscriptomes among patients with COVID-19 disease
Source: Sci Rep. 2022 Dec 7;12:21125. doi: 10.1038/s41598-022-25463-0 (PMC9729217; doi:10.1038/s41598-022-25463-0)
Supplement: Supplementary file 18 — Supplementary Legends. [file 41598_2022_25463_MOESM18_ESM.docx]

**Supplementary Information**

Supplementary Table 1: COVID-19 BALF Data Sources

Supplementary Table 2: Non-COVID-19 BALF Data Sources

Supplementary Table 3: Statistically Significant GO Terms among COVID-19 vs. CAP vs. Uninfected

Supplementary Table 4: Statistically Significant GO Terms among COVID-19 Survived vs. Deceased

Supplementary Table 5: Dirichlet Multinomial Mixtures (DMM) Clusters

Supplementary Table 6: Statistically Significant Taxons among COVID-19 vs. CAP vs. Uninfected

Supplementary Table 7: Diversity Metrics

Supplementary Table 8: Statistically Significant Taxons among COVID-19 Survived vs. Deceased

Supplementary Table 9: List of Proteins Associated with GO:0000287 – Magnesium Ion Binding

Supplementary Table 10: List of Proteins Associated with GO:0003723 – RNA Binding

Supplementary Table 11: List of Proteins Associated with GO:0004175 – Endopeptidase Activity

Supplementary Table 12: List of Proteins Associated with GO:0006310 – DNA Recombination

Supplementary Table 13: List of Proteins Associated with GO:0008270 – Zinc Ion Binding

Supplementary Table 14: List of Proteins Associated with GO:0016491 – Oxidoreductase Activity

Supplementary Table 15: List of Proteins Associated with GO:0034654 – Nucleobase Containing

Compound Biosynthetic Process

Supplementary Table 16: List of Proteins Associated with GO:0072528 – Pyrimidine Containing

Compound Biosynthetic Process

Supplementary Table 17: List of Proteins Associated with GO:1901565 – Organonitrogen Compound

Catabolic Process

Supplementary Table 18: Supplementary Table Legends
